# A scoping review of children’s and adolescents’ active travel in China

**DOI:** 10.3389/fpubh.2026.1771411

**Published:** 2026-08-05

**Authors:** Fanxing Kong

**Affiliations:** School of Physical Education, Suihua University, Suihua, Heilongjiang, China

**Keywords:** active travel, children, China, physical activity epidemiology, scoping review

## Abstract

**Background/objective:**

Active travel (AT) is a frequent and feasible source of daily physical activity for school-aged youth; however, no comprehensive synthesis has mapped the evidence for China. This scoping review aimed to characterize the current research landscape on AT among children and adolescents in China and identify gaps to guide future research and policy.

**Methods:**

This study searched five databases (Web of Science, PubMed, SPORTDiscus, MEDLINE, and ERIC) from inception to 1 December 2025. Quantitative studies in English that examined AT among Chinese children and adolescents were included. The reviewer screened records, extracted data, and summarized study characteristics, measures, and topics.

**Results:**

Of the 6,178 records screened, 16 met the inclusion criteria. Publications have increased notably in recent years, with 75.0% appearing from 2017 to 2023. The majority of studies were cross-sectional (93.8%), and one was longitudinal (6.3%). Travel behavior was predominantly self-reported (87.5%), with device-based measures used to capture activity in 12.5% of studies, and none of the studies used devices to capture travel modes (0%). Studies primarily focused on school-aged children/adolescents in urban settings (81.3%), with a minority spanning mixed urban–rural samples (18.8%). By topic, 56.3% examined correlates, 43.8% assessed health or physical activity outcomes, and 25.0% reported AT prevalence.

**Conclusion:**

Research on AT in China is growing; however, it remains dominated by cross-sectional, self-reported studies, highlighting the need for stronger designs and measures to generate decision-ready evidence. Future studies should prioritize standardized, device-based measures, longitudinal or quasi-experimental designs, and rigorous evaluations of multicomponent and infrastructure interventions that have proven effective in increasing school-based AT. Equity-focused sampling across urban–rural contexts and broader health outcomes (including mental health) is also needed, while leveraging established individual, family, and built-environment correlates to guide policy and practice in China. Moreover, mixed findings on fitness benefits suggest that careful outcome selection and a robust causal design are essential.

## 1 Introduction

Regular engagement in physical activity (PA) is beneficial for various health indicators in children and adolescents, such as healthy weight status, improved physical fitness, enhanced academic performance, and mental health. Participating in PA can be accomplished through multiple modalities, such as active travel (AT), sports participation, and physical education. AT is considered a relatively feasible approach, as it occurs more frequently than other forms of PA (1, 2) and is associated with some physical (3–5) and mental health (or illness) outcomes (1, 2, 5, 6).

Owing to the lower levels of PA in children and adolescents, finding more opportunities to increase PA levels is of great importance. In this respect, AT is an essential source, as it is associated with higher levels of PA (7). However, the levels of AT in children and adolescents worldwide are disappointing. For example, the Global Report Card Project (v3.0) data shows that the level of AT in children and adolescents worldwide is rated as C, suggesting that approximately half of this population has regular AT behavior (8). Another international collaborative study (sample size = 277,833 adolescents) indicated that the prevalence of AT was 45.1% (7). These data show that the levels of AT in children and adolescents should be elevated from the perspective of health promotion. Regarding a specific country, the situation is not ideal. In the US, only 9.6% of students aged between 5 and 17 years usually walk, and 1.1% biked to school (9). Comparatively, 39.9% of Chinese children walked, and 15.9% cycled to school, while 44.2% traveled by passive means (10). Taken together, it is likely that children and adolescents are insufficiently engaged in AT.

To the author’s knowledge, no study has comprehensively summarized the evidence on children’s and adolescents’ AT in China. Existing reviews on youth AT have mapped environmental and behavioral determinants, documented long-term shifts toward motorized commuting, and synthesized links with physical activity; however, the evidence remains fragmented and context-dependent (11, 12). A recent scoping review in Ireland identified 19 studies and highlighted heterogeneity in measures and a limited evaluation of mental well-being outcomes, underscoring the need for country-specific syntheses that reflect local policy and urban form (13). Global intervention reviews show that well-designed school-based programs, especially those supporting cycling, can shift mode share; however, effectiveness varies with implementation, infrastructure, and parental safety perceptions (14, 15). Ultimately, this scoping review has the potential to inform policymakers, practitioners, and researchers in order to delineate programs and strategies for promoting active travel among children and adolescents in China. This scoping review aimed to characterize the current research landscape on AT among children and adolescents in China and identify gaps to guide future research and policy.

## 2 Methods

As the research aim of this scoping review was to map and summarize the current status of research on active travel in Chinese children and adolescents, this scoping review was in line with the established scoping review methodological framework developed by Arksey and O’Malley (16). The specific procedures were as follows:

- Stage 1. Identifying the research question,
- Stage 2. Searching for relevant studies,
- Stage 3. Selection of studies,
- Stage 4. Charting of data,
- Stage 5. Collating, summarizing, and reporting the results.

### 2.1 Identifying the research question

To the author’s knowledge, no review has been published that comprehensively summarizes the evidence on children’s and adolescents’ active travel in China. This raised the following research question: “What is the nature and content of research on active travel of children and adolescents conducted in the Chinese context, and what type of implications are (not) being addressed?” Therefore, the purpose of this scoping review is to (a) map and summarize the existing literature findings, specifically relating to contextual study factors, such as age and gender, for active travel in children and adolescents from China; (b) document the existing study design, measurement protocol, and prevalence of active travel in these identified scoping reviews; and (c) identify current barriers, gaps, and areas of opportunity for active travel promotion in the literature.

### 2.2 Searching for relevant studies

Before the literature search, the research questions and search strategy were refined. Searches were then conducted in five electronic databases (WOS, PubMed, SPORTDiscus, MEDLINE, and ERIC) on 1 December 2025. The search strategy consisted of two sets of terms (two sets were combined with “AND” and applied to article titles): (1) (“China” OR “Chinese”), and (2) (“walk” OR “walking” OR “run*” OR “running” OR “bike” OR “biking” OR “bicycle” OR “bicycling” OR “cycle” OR “cycling” OR “stroll” OR “strolling” OR “ride” OR “riding” OR “active transport” OR “active transportation” OR “active transit” OR “active transporting” OR “active commut*” OR “travel mode” OR “mode of travel*” OR “active travel” OR “active travelling” OR “trip” OR “school travel” OR “non-motorized”). Reference lists of included articles were screened for additional peer-reviewed studies; gray literature was not included in this version. This scoping review was conducted in accordance with the PRISMA-ScR; the PRISMA-ScR checklist (Supplementary material S1) and full database-specific search strategies (Supplementary material S2) are provided.

### 2.3 Selection of studies

This study conducted a literature search, merged records, and removed duplicates. All records from the literature search were imported into EndNote X9 software. After removing duplicates, the remaining records were screened by a reviewer in terms of their titles and abstracts. Studies were included in the present review if they (1) included populations that were typical children and adolescents aged 5–17 years in China; (2) conducted research on AT; (3) collected any quantitative data related to AT, including levels, prevalence, correlates, determinants, outcomes, measurement tools, or intervention (intervention trials in the Chinese population in China); (4) included any type of AT measure, such as self-reports or device-based measures; and (5) were published as peer-reviewed articles in English journals. Studies were excluded if they (1) targeted non-Chinese populations and Chinese people outside of China; (2) focused on special children and adolescents (condition, disease, or disability); or (3) were published as a literature review, commentary, conference abstract, or editorial. A single reviewer screened titles/abstracts and extracted data; an independent colleague verified a 20% random subsample to minimize selection/extraction bias.

### 2.4 Charting of data and collating, summarizing, and reporting the results

The data of the included studies were compiled according to the following data extraction method, and descriptive analysis was performed. The following data were extracted from the included studies: (1) author names and publication year; (2) study design and study site; (3) sample; (4) study measure; (5) content of AT; and (6) study findings. Table 1 shows the detailed charting dimensions and guidelines for each study.

**Table 1:** Charting dimensions and guidelines.

Charting dimension	Charting guideline
Study design/study site	Refer to if research was an intervention, a multiple baseline case study, a cross-sectional study, etc., and the sample source.
Sample	Refer to if the sample age, sample size, and the percentage of boys.
Active travel content	Identify the contents included (e.g., AT patterns, active school travel modes, and AT behaviors).
Measures	Refer to if data were self-reported or objectively measured with specific tools, if relevant.
Summary of findings	Summarize data only related to active travel, including the prevalence and determinants/correlates

## 3 Results

**Figure 1:**
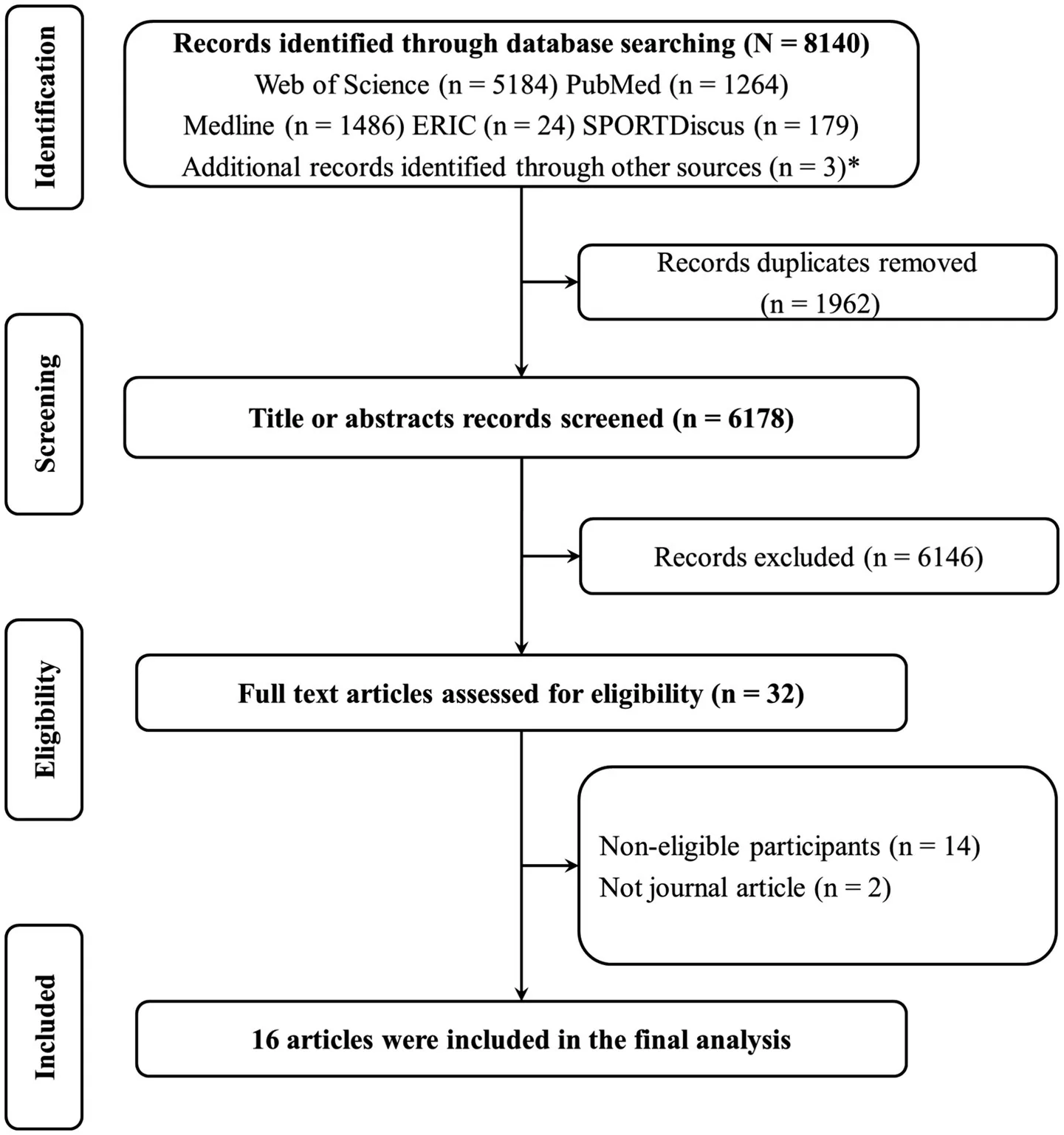
Flow diagram of the study selection process. *, Article with references to one included study.

An overview of the study selection of this scoping review is displayed in accordance with the PRISMA-ScR (17), as shown in Figure 1. A total of 8,140 records were identified through Web of Science, PubMed, MEDLINE, ERIC, and SPORTDiscus, with three additional records from other sources. After removing 1962 duplicates, 6,178 records were screened by title and abstract, and 6,146 were subsequently excluded. A total of 32 articles underwent full-text assessment. Of this, 14 were excluded because of non-eligible participants, and 2 were excluded because they were not peer-reviewed journal articles. A total of 16 articles were included in the final analysis (1, 10, 18–31).

### 3.1 Overall characteristics

**Figure 2:**
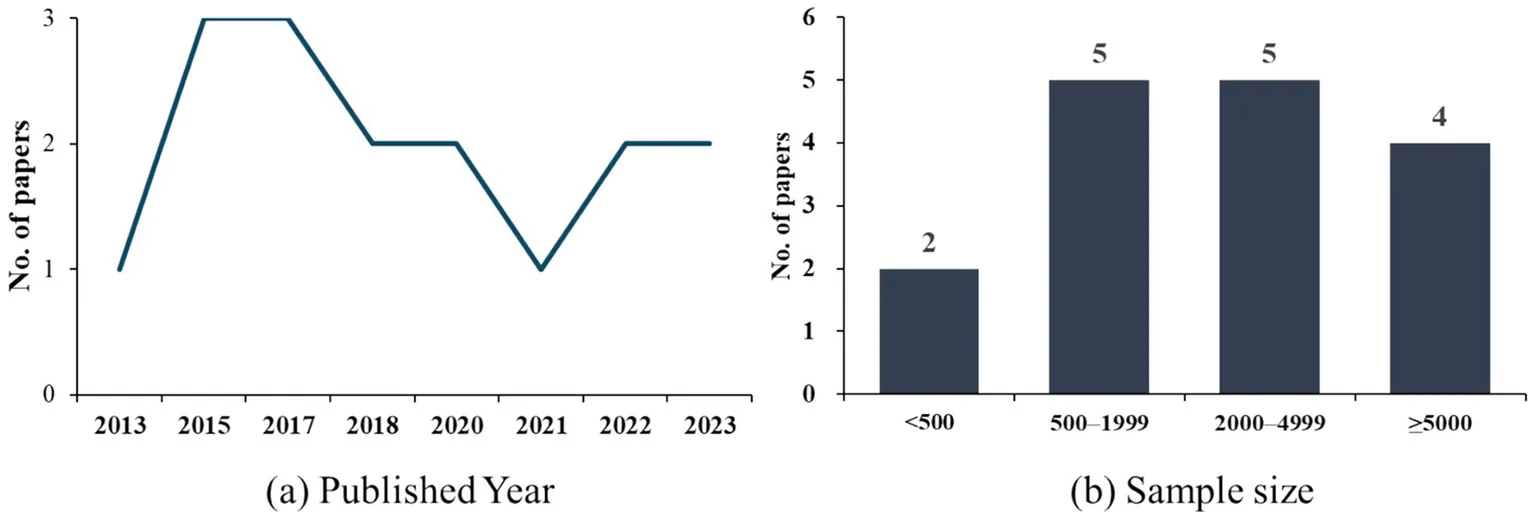
Characteristics information in the published year and sample size.

Of the 16 included studies (1, 10, 18–31) published between 2013 and 2023, the study count fluctuated, peaking at three in 2015 and 2017 and stabilizing at two per year in 2020, 2022, and 2023 (Figure 1 and Table 2). There were 15 cross-sectional studies and one longitudinal study (1997–2011) covering major cities and provinces in mainland China (e.g., Beijing, Shanghai, Shenzhen, Guangzhou, Shenyang, Wuhan, Hefei, Zhengzhou, Chongqing, Kunming, Tianjin, Xi’an, Wenzhou, Ganzhou, Fuyang, Tongzhou, and provinces including Liaoning, Jiangsu, Shandong, Henan, Hubei, Hunan, Guangxi, Guizhou, and Heilongjiang) and Hong Kong, with one two-site comparison including Nanjing (China) and Mashhad (Iran). The total sample size was approximately 68,868 participants, with per-study samples ranging from 68 to 21,596. The four categories of sample size shown in Figure 2b contain “<500” (*n* = 2), “500–1999” (*n* = 5), “2000–4,999” (*n* = 5), and “≥5,000” (*n* = 4), respectively. Seven studies included both children and adolescents, five studies included children only, and four studies included adolescents only (see Figure 2b).

**Table 2:** Summary of reviewed articles.

Study (year)	Study design (study site)	Sample	Active travel	Measures	Study findings
Quan (2013) (18)	Cross-sectional (Chengdu, Fuyang, Ganzhou, Guangzhou, Shanghai, Shenyang, Tianjin, Tongzhou, Wenzhou, Xi’an, and Yingtan)	Age range: 9–17, *n* = 2,751, boys: 51.4%	Walking behavior	ActiGraph GT3X accelerometers	(1) Participants walked more steps during weekdays than weekend days (*p* < 0.01); the most active walking day was Friday. (2) Boys walked more steps than girls (*p* < 0.01). (3) The most active age was 11 years in boys and 10 years in girls, and older children walked fewer steps. (4) The data suggest that a majority of the Chinese city children and youth did not meet the recommended health-related steps/day.
Gao (2015) (19)	Cross-sectional (Hong Kong)	Age range: 10–11, *n* = 68, boys: 41.2%	Pedometer-determined PA patterns	YAMAX pedometers	(1) The top segments contributing to total daily steps were steps after school (34.2%). (2) Boys were more active than girls in stepping out of school (*p* < 0.05). (3) Students accumulated 914 more steps on days that included PE classes than on days without PE classes.
Li (2015) (20)	Cross-sectional (Beijing)	Age range: 13–15, *n* = 3,267, boys: 51.1%	Commuting mode choice: walk, bicycle, public transport, car, other	Self-reported questionnaire: travel distance, travel time, travel origin and destination, travel modes	(1) Students who live in the suburban areas travel longer distances than those who live in the core center of the city, where the majority of the good-quality schools are located. (2) In the core area, for students whose school is within 3 km of home, cycling is more popular than other modes. For students who must travel further than 3 km, there is a significant modal split between households with different socioeconomic statuses and car ownership.
Sun (2015) (10)	Cross-sectional (Shenyang, Shanghai, Hefei, Wuhan, Zhengzhou, Chongqing, Kunming, Guangzhou)	Grade: 4–12, *n* = 21,596, boys: 43.7%	Active school travel	Self-reported questionnaire: In the last 7 days, how did you usually get to your school?	(1) A total of 39.9% of Chinese children walked, 15.9% cycled to school, and 44.2% traveled by passive commuting. (2) Active school travel was associated with 0.855 lower odds of being obese (*p* < 0.001) and 0.907 lower (*p* < 0.001) odds of having depressive symptoms compared with children using passive transport.
Huang (2017) (21)	Cross-sectional (Hong Kong)	Age range: 7–10, *n* = 677, boys: 56.0%	Active school travel	Self-reported questionnaire 1: How many times does your child usually walk to school in a typical week? 2: How many times does your child usually walk from school in a typical week?	(1) On average, a single home–school journey lasted for 10 min. (2) Compared with children who consistently used passive travel modes, a change from passive to active travel to school was positively associated with changes in the percentage of time spent in MVPA and MVPA min/day on weekdays. Similar results were found for weekly MVPA.
Yang (2017) (22)	Longitudinal study (Liaoning, Jiangsu, Shandong, Henan, Hubei, Hunan, Guangxi, Guizhou, Heilongjiang, Beijing, Shanghai, and Chongqing)	Age range: 6–17, *n* = 9,487, boys: 52.9%	Active school travel	Self-reported questionnaire 1: Do you travel to and from school this way? 2: How long does a round trip take?	(1) The prevalence of reported active school travel among children dropped from 95.8% in 1997 to 69.3% in 2011. (2) Active school travel was common in children living closer to school, in middle school, from low-income households, with low parental education status, and those without a private vehicle (*p* < 0.05). Children who were living in a metropolitan area and who had more than 40 min of total PA per day were less likely to report active school travel (*p* < 0.05).
Zhang (2017) (24)	Cross-sectional (Beijing)	Age range: 7–18, *n* = 4,580, boys: 52.0%	School travel mode choice: walk, car, bus or subway, cycle, or other modes	Self-reported questionnaire: travel purpose, travel origin and destination, travel mode, travel time, departure time	(1) Car ownership, poor walking/cycling environment, and adults’ convenience for escorting students significantly stimulate the use of cars in school commuting. (2) Students are more inclined to choose cars when their departure time is during rush hour compared to other times. (3) The longer distance encourages the use of motorized transport, and households with local hukou (usually related to car ownership and other welfare) are more willing to drive their children to school.
Meng (2018) (25)	Cross-sectional (Shenzhen)	Age range: 12–15, *n* = 1,257	Active school travel	Students completed an in-class chart for each day and hour and traced their route on an attached map	Features of the route environment are related to both travel mode and active travel route choice.
Sun (2018) (26)	Cross-sectional (Shenzhen)	Age range: 9–14, *n* = 805, boys: 52.0%	Active commuting behaviors	(1) Self-reported travel mode: walking, bicycling, automobile, public transit, and other (2) Self-reported daily travel time: less than 5 min, 5–10 min, 10–15 min, and greater than 15 min. (3) Asked subjects if they had different routes to and from school: the same, totally different, or partially different. (4) Commuting companionship: alone, classmates or friends, parents, grandparents, or nannies.	(1) Three out of four children lived within their school catchment neighborhoods, of which 87% were active commuters. For children living outside of school catchment areas, 22% walked or cycled to school. (2) Having more gates, measured by the density of neighborhood entrances, was positively associated with active mode choice. Route aesthetics greatly impacted the active mode choice. Safety was associated with different routes used to and from school. Compared to children who commuted alone, children who traveled with classmates or parents were less likely to use different routes to and from school.
Gu (2020) (1)	Cross-sectional (Shanghai)	Age: 13.7 ± 0.9, *n* = 6,478, boys: 53.8%	Active school travel	Question: How many days did you walk or ride a bicycle to and from school during the past 7 days? Item: 0-7 day days	(1) 53.2% of adolescents reported being active in school travel without sex difference. (2) Active school travel was associated with reporting no depressive symptoms in adolescents (adjusted OR = 1.20, 95%CI: 1.06–1.36). (3) The relationship was significant in boys (adjusted OR = 1.34, 95%CI: 1.11–1.60) and those in grades 8 (adjusted OR = 1.25, 95%CI: 1.01–1.55) and 9 (adjusted OR = 1.29, 95%CI: 1.01–1.65).
Fallah Zavareh (2020) (47)	Cross-sectional (Mashhad in Iran, Nanjing in China)	Age range:7–9, *n* = 849 (Iran 625; China 224)	Mode of travel	Questionnaires for parents: Report the mode of travel that is most frequently used when the child normally commutes to school.	(1) Walking time from home to school and parental worry about road crashes were negatively associated with children walking to school in both samples.
Xiao (2021) (27)	Cross-sectional (Shenzhen)	Age: Grade:1–6, *n* = 1970	School travel	Questionnaire: This included departure time in the morning from home to school, duration, travel distance, mode of transportation, chauffeuring/pickup, departure time, and arrival time from school to home.	(1) Students without a legitimate hukou to local areas face more constraints, longer distances, and more time to access private schools, which are excluded from public sponsorship and have disadvantages in terms of geographical locations. (2) Household vehicle ownership and travel features (i.e., chauffeuring and home-school distance) have a much more significant role in school travel mode decisions, which largely surpasses the role of individual demographic attributes and the school’s surrounding built environment play.
Ding (2022) (28)	Cross-sectional (29 county-level units in mainland China)	Age: 8th-grade, *n* = 4,807	School travel	Question	(1) Commute times are significantly negatively associated with children’s PWB and academic achievements, and this correlation varies across areas. (2) Children living in the urban fringe have the longest average one-way commuting time (18.6 min); however, they have a better acceptance of longer commuting duration, whereas commuting time is more influential in the city center and rural areas. (3) Regarding travel mode, walking to school is positively associated with PWB in the central area, whereas bicycles and public transport positively affect the rural student scores.
Wang (29, 34)	Cross-sectional (Beijing)	Age: 9.04 ± 1.83, *n* = 3,447	Walking to school	Self-reported: Subjects could choose from 14 response items, including walking, car, motorbike, cycling, bus, metro, etc.	(1) Schoolchildren, especially those from higher-income families, are more likely to walk to school when they live in neighborhoods with lower street walkability, whereas higher street enclosures were associated with higher odds of walking for all respondents. (2) A non-linear but overall negative relationship was observed between the odds of walking and street safety facilities. (3) Schoolchildren’s household car ownership, educational attainment of parent(s), and household annual income modify the built environment-walking to school association.
Ding (2023) (30)	Cross-sectional (29 county-level units in mainland China)	Age: 14.03 ± 0.79, *n* = 6,353	School travel mode	The question was, ‘What mode do you usually use from home to school?’	Students who biked to school had a higher self-rated health status, healthier weight, lower level of mental stress, and lower risk of developing brain diseases.
Wang (2023) (31, 41)	Cross-sectional (Guangzhou)	Age: 12–18, *n* = 476	Activity places and active travel patterns	Respondents reported their activities and trips within 24 h in chronological order in the activity and travel diary.	(1) Streetscape greenery was negatively associated with the odds of AT and positively associated with the number of AT trips on weekdays and negatively related to AT duration on weekends. (2) Pavement ratio and traffic volume were negatively associated with the odds of AT and AT duration on weekdays. (3) Safety facilities and street vitality were positively associated with AT frequency and duration on weekends and weekdays. (4) Gender is an effect modifier of the built environment-AT pattern associations.

PA, physical activity; PE, physical education; AT, active travel; MVPA, moderate-to-vigorous physical activity; OR, odds ratio; CI, confidence interval.

### 3.2 Methodological characteristics of the included studies

Table 2 illustrates that the majority of studies (*n* = 14) used self- or parent-reported items to capture usual travel mode (e.g., walking, cycling, bus/metro, car, motorbike, public transit, other), frequency (e.g., days active in the past 7 days), one-way or round-trip duration, home–school distance (e.g., ≤3 km vs. >3 km), departure time (including rush hour), school catchment status, chauffeuring/pickup, companionship (alone, classmates/friends, parents, grandparents/nannies), and whether routes differed to/from school; some captured simple mental health or well-being items. Two device-based studies used accelerometers (ActiGraph GT3X) or pedometers (YAMAX) to quantify steps and segmental activity patterns; two used travel diaries or in-class mapping to trace routes and 24-h trips. Built-environment features included the density of neighborhood entrances (gates), streetscape greenery, street walkability/enclosure, pavement ratio, safety facilities, traffic volume, street vitality, route aesthetics, and perceived safety; individual/household covariates commonly included vehicle ownership, hukou, and parental education/income.

### 3.3 Main findings of the included studies

#### 3.3.1 Prevalence, modes, and patterns

As shown in Table 3, seven studies reported the prevalence, modes, or patterns. In a multicity sample, 39.9% of children walked, 15.9% cycled to school, and 44.2% used passive modes; in an urban adolescent cohort, 53.2% reported active school travel (AST). Nationally, the reported active travel prevalence declined from 95.8% (1997) to 69.3% (2011). Distance and catchment status were pivotal; in Beijing’s core area, cycling was more popular when the school distance was ≤3 km. Beyond 3 km, modal splits differed by socioeconomic status and car ownership. In Shenzhen, three-quarters of students lived within school catchment areas, and 87% of them used active modes; among those living outside the catchment, 22% walked or cycled. A single home–school journey averaged 10 min in Hong Kong, and 8th-grade students living in the urban fringe had the longest average one-way commute (18.6 min). Weekday steps exceeded weekend steps, with Friday being the most active day. Steps after school contributed 34.2% of daily steps, and days with physical education yielded 914 more steps than days without (Table 2).

**Table 3:** Research areas of active travel.

Research area	Authors (year)	No.
Prevalence/patterns	Yang (2017) (22), Sun (2015) (10); Gu (2020) (1); Sun (2018) (26); Quan (2013) (18); Gao (2015) (19); Huang (2017) (21)	7
Correlates/determinants*		
*Individual (age/sex/residence)*	Quan (2013) (18); Yang (2017) (22); Xiao (2021) (27); Wang (2023) (31, 41)	4
*Environment*		
Built and natural	Li (2015) (20); Zhang (2017) (24); Meng (2018) (25); Sun (2018) (26); Wang (2022) (29, 34); Wang (2023) (31, 41); Yang (2017) (22); Fallah Zavareh (2020) (47); Xiao (2021) (27); Ding (2022) (28)	10
*Social*		
School	Gao (2015) (19); Sun (2018) (26); Xiao (2021) (27); Yang (2017) (22); Li (2015) (20)	5
Family/parenting	Li (2015) (20); Yang (2017) (22); Zhang (2017) (24); Sun (2018) (26); Fallah Zavareh (2020) (47); Xiao (2021) (27); Wang (29, 34)	7
Other	Zhang (2017) (24)	1
Outcomes		
*Psychosocial*		
Depressive symptoms	Sun (2015) (10); Gu (2020) (1)	2
Well-being	Ding (2022) (28)	1
Stress	Ding (2023) (30)	1
Academic achievement	Ding (2022) (28)	1
*Behavioral and health*		
Behavioral	Quan (2013) (18)	1
Adiposity	Sun (2015) (10); Ding (2023) (30)	2
Health risk	Ding (2023) (30)	1

*, Socio-ecological framework.

#### 3.3.2 Correlates and determinants

As shown in Tables 2, 3, built/natural environmental correlates were most frequently examined (*n* = 10), followed by social domains—school (*n* = 5) and family/parenting (*n* = 7)—with individual factors (age/sex/residence; *n* = 4) and other factors (*n* = 1) being less common. Shorter home–school distances and more walkable or rideable routes were consistently associated with higher odds of walking or cycling, whereas car ownership, hukou constraints, and lower active mode use. Built-environment attributes—street enclosure, gate density, safety facilities, greenery, pavement ratio, traffic volume, street vitality, and route aesthetics—showed mode-specific and area-specific associations, with evidence of moderation by household socioeconomic status and gender. Departure at rush hour increased car use, and school siting/catchment access shaped feasible mode choices.

#### 3.3.3 Outcomes

Psychosocial outcomes were assessed in five studies: depressive symptoms (*n* = 2), well-being (*n* = 1), stress (*n* = 1), and academic achievement (*n* = 1). Active school travel was associated with fewer depressive symptoms, walking in urban centers was positively related to psychological well-being, and cycling was linked to lower stress. Longer commutes were negatively associated with psychological well-being and academic achievement, and area-specific differences (bicycles/public transport) were positively associated with rural students’ academic scores. Behavioral and health outcomes included behavioral measures (*n* = 1), adiposity (*n* = 2), and health risks (*n* = 1). Switching from passive to active modes was associated with higher MVPA; adolescents who biked reported healthier weight and higher self-rated health, and active travel was linked to healthier adiposity profiles.

## 4 Discussion

This scoping review aimed to map and summarize existing studies on AT in Chinese children and adolescents. Based on the prior analysis, this study identified current points relevant to the research status and future development. The discussion is presented in the following order: (1) main research findings; (2) method characteristics; and (3) current research gaps and future research directions. Detailed analytical content is presented below.

### 4.1 Main research findings

This scoping review found that over the past few years, there have been a few studies (a limited number: 16) that focused on active travel in children and adolescents in China. Unlike other reviews of studies related to physical activity among Chinese populations or subpopulations, the limited number of studies that focused on children’s and adolescents’ AT in China implies insufficient attention to this behavior. Also, compared with the scoping review of children’s and adolescents’ AT in Ireland (13), this study included only 16 studies, while the former one included 19 studies (published 2000–2020). This comparison indicates less attention to studies of AT in China. However, because of the health benefits (e.g., physical, mental, behavioral) of AT, it is recommended that interested or relevant researchers focus on studies of AT, which provide China-based empirical evidence to advance the knowledge in this field and offer more effective practical implications. These mobility effects matter for health: systematic evidence shows that active school travel is consistently associated with better cardiorespiratory fitness in youth, and commute time itself carries psychosocial impacts (longer commutes are linked to poorer psychological well-being and lower academic performance); together, these channels justify treating active school travel as a priority for child health policy, not only for transport planning. Although an increasing number of studies have focused on children’s and adolescents’ AT, conducting studies based on a theoretical framework is worth mentioning. As AT is a component of physical activity, frameworks to study physical activity are also applied to studies of AT. To the author’s knowledge, physical activity epidemiology and the growing socio-ecological literature situate AT within multilevel determinants (individual, parental, household, school, and built environment), which should guide the design of future China-based studies and interventions.

### 4.2 Trends and contributions of method characteristics in China

A concise international comparison indicates that the percentages observed in this review (40% walking, 16% cycling, and 50% any active school travel) are higher than those in many Anglophone contexts but lower than the highest European benchmarks. In the United States, ATS is generally lower and declines sharply with distance, with car access and safety concerns shifting families toward motorized modes (9), while cross-country tracking shows large variation in walking (e.g., 55% in the Czech Republic vs. 30% in Wales), underscoring contextual effects. Australia has also recorded continued declines in active school travel over recent years (32). Mechanistically, shorter home–school distances and school siting that keeps catchments local favor walking and cycling, whereas longer trips push households toward passive modes (33); built-environment and safety factors (street connectivity, traffic exposure, perceptions) independently shape mode choice, and household transport options amplify socioeconomic differences in modal splits (9). These drivers align with the findings of high active shares within catchments and lower active use among students living farther away or in fringe areas.

Methodologically, measurement was dominated by self- or parent-report (14 of 16 studies), with only two device-based studies quantifying activity via accelerometers or pedometers, and no studies using devices to capture travel mode. Two studies complemented questionnaires with travel diaries or in-class route mapping. Built-environment exposures were examined in 10 studies—most often in large urban settings—and included street enclosure and walkability, safety facilities, greenery, pavement ratio, traffic volume, street vitality, and neighborhood gate density, alongside perceived safety and route aesthetics. Individual and household covariates frequently included age/sex, vehicle ownership, hukou status, chauffeuring, and departure time. To balance feasibility and accuracy across urban and rural contexts, future studies should adopt tiered measurement: (a) urban sites, smartphone GPS logging linked to a 7-day travel diary; (b) rural/low resource-sites, pedometers plus teacher/parent-verified travel diaries with map-based recall and school-gate observations. A harmonized minimal dataset should classify trips at the segment level (e.g., walk-to-transit) and record mode, duration, distance, companions, and peak-period status.

Consistent correlates and determinants emerged across sites. Shorter home–school distance/time was a primary positive predictor of walking and cycling, and car ownership, hukou constraints, chauffeuring, and rush-hour departures lowered active mode use. Safer traffic environments, better connectivity, and supportive route features were associated with higher AT, with evidence of moderation by household socioeconomic resources and gender (34, 35). School siting and catchment access shaped feasible mode choices, reinforcing the role of institutional structures. These China-based patterns align with broader evidence indicating that built environment upgrades (connectivity, safe crossings, and speed management) can increase AT when they align with parental assessments of safety and convenience, underscoring the central role of parental perceptions and licensing in children’s mode choice (36–39). Beyond city case studies, national panel analyses that combine child, parent, household, and environment variables within a single framework further clarify socio-ecological influences (40). Internationally, declines in walking and cycling to school have been documented with increasing urbanization and motorization progress, further motivating systematic monitoring and support for AT in China. The evidence base is broad and largely cross-sectional, with scalable self- or parent-report for mode, frequency, duration, distance, and companionship, complemented by device measures and travel diaries in a minority of studies; segment-specific results show clear after-school and physical education day contributions to daily activity, clarifying when AT yields the most benefit.

Outcomes evidence, although limited, points to important psychosocial and health links. Active school travel was associated with fewer depressive symptoms; walking in urban centers was positively related to psychological well-being; and cycling was linked to lower stress. Longer commutes were negatively associated with psychological well-being and academic achievement, with area-specific differences (e.g., bicycles/public transport positively associated with rural students’ academic scores). Behavioral and health outcomes showed that switching from passive to active modes was associated with higher MVPA, adolescents who biked reported healthier weight and higher self-rated health, and active travel was linked to healthier adiposity profiles. Given the predominance of cross-sectional designs, these associations should be interpreted cautiously; however, they substantively align with the notion that AT contributes to movement behaviors and selected health indicators.

Built environment quality shapes both actual behavior and adolescents’ perceptions, and those perceptions mediate behavior: safer, greener, and more engaging streets are linked to higher odds, frequency, and duration of active school travel, with adolescents’ environmental perceptions transmitting built-environment effects to AT (and, where AT increases, to better subjective well-being) (41). This mediation pathway explains why neighborhoods with similar infrastructure can yield different AT rates if one elicits stronger feelings of safety and comfort among youth, and it underscores the need for continuous, protected walking/cycling networks—rather than isolated improvements—to build route confidence and parental licensing (41). Chinese research increasingly integrates streetscape metrics (greenery, safety facilities, vitality, and connectivity) with route perceptions to generate context-sensitive insights for high-density urban forms, while national socio-ecological evidence shows multifaceted influences—distance, road network density, temperature, household resources, and parental characteristics—shaping AT across ages and places (42). These findings justify combining objective environmental measures with subjective perceptions in analysis and policy so that interventions can be tailored to both physical route quality and how young people experience it on the ground (41).

### 4.3 Current research gaps and future research directions

Key gaps remain in longitudinal and intervention designs to establish causality and quantify health gains from sustained AT, particularly across transitions from primary to secondary school. Objective mobility measurements (e.g., GPS-linked accelerometry) are underused and would better capture multimodal and partially active trips (e.g., walk-to-transit) common in Chinese cities (43). Social environment constructs—independent mobility licensing, peer accompaniment, and parental safety perceptions—need deeper measurement, as international evidence shows that perceived safety, distance, and parental gatekeeping strongly shape children’s mode choices (42). Preschool and early-primary cohorts are understudied, even though distance and parental worries are already influential at these ages (44).

The mapped evidence suggests unequal access to active school travel, with much higher active shares within catchments, divergence by socioeconomic status and car ownership beyond 3 km, and the longest commutes in urban fringe areas. Future studies should assess the distributional impacts of school siting and route safety, stratify by socioeconomic indicators and car access, and evaluate changes that shorten trips and improve safe, direct routes. Mental health outcomes were not directly measured in the included studies; therefore, subsequent research should add validated well-being and stress measures alongside travel and activity indicators and examine whether associations differ by distance, catchment status, and after-school periods.

This study recommends longitudinal cohorts anchored at key educational transitions (school entry at grade 1; primary-to-lower secondary at grades 6–7; lower-to-upper secondary at grades 9–10) and at household relocation or catchment changes to track mode shifts and health outcomes. Attrition can be mitigated through multi-channel contact updates, school-based coordination, modest incentives, and predefined imputation plans. Policy shocks (e.g., homework and after-school policy adjustments and traffic-calming initiatives) should be modeled as time-varying covariates, using stratified analyses and difference-in-differences where appropriate. Core outcomes for Chinese children and adolescents should include cardiorespiratory fitness (20-m shuttle run), device-based movement behavior, and mental health [Patient Health Questionnaire–Adolescent version (PHQ–A) and brief stress scales]. Standardized, school-based protocols with quality control are recommended to enhance comparability.

For interventions, feasible pathways in China include Safe Routes to School (traffic calming, crossings, and wayfinding), walking school buses, bicycle skills training with secure parking, and parent engagement to improve perceived safety. Typical durations of 12–24 weeks are appropriate. Core evaluation indicators should include mode share, trip frequency and duration, perceived safety, independent mobility licensing, and school-gate traffic, assessed using controlled before–and–after, difference-in-differences, or cluster-randomized designs adjusted for school type, household socioeconomic status, catchment status, and concurrent policies. Finally, this study’s finding of insufficient rural coverage supports multi-stage stratified sampling by urban–rural status, region, and economic level, with targeted inclusion of migrant and left-behind children to enable subgroup-specific analyses of determinants.

### 4.4 Policy and practice implications

Planning and policy should prioritize shortening home–school distances (e.g., catchment-based siting and enrollment), continuous and protected walking/cycling networks, and safe crossings to remove the strongest barrier to AT (42). Route-level design that enhances greenery, safety facilities, and street vitality appears to increase the odds and amount of active travel and should be tailored to local urban forms (core, fringe, and mountainous) (40). Safe Routes to School programs, walking school buses, and bicycle skills training can counter perceived safety barriers and embed daily movement into routines, while school and community initiatives that strengthen adolescents’ positive route perceptions may boost both AT and well-being (41, 43). Targeted strategies for high-income, car-owning households are warranted, as these groups show lower AT; policies that improve the convenience and perceived safety of active modes and manage school-gate traffic could shift behavior (43). Familiarizing children and adolescents with walking and cycling routines (e.g., supervised walking school buses and age-appropriate bicycle skills curricula) reduces perceived barriers and supports mode shifts. Institutional structures uniquely shape feasible options in this context: enrollment rules and catchment access influence home-to-school distance, escorting, and chauffeuring, while pronounced peak-period congestion elevates car use; these patterns imply that neighborhood design cannot substitute for proximity created by school siting and admissions, and staggered start times, crossing guards, and traffic calming around school precincts should complement streetscape improvements.

### 4.5 Study strengths and limitations

This is the first scoping review to map evidence on AT among Chinese students using a socio-ecological model. Several limitations should be noted. First, the review was restricted to peer-reviewed journal articles in English-language, which likely omitted relevant Chinese-language studies and may reduce representativeness. This restriction was intended to maximize reporting consistency and international reproducibility. Because publication language can correlate with differences in study populations and risk of bias. Including Chinese-language studies could affect precision and possibly some estimates (45); at the same time, comparative meta-analyses of English- and Chinese-language literatures sometimes report convergent effect patterns (46). This study did not search Chinese-language databases or conduct a language-sensitivity analysis; therefore, the impact of this restriction on the conclusions cannot be quantified here. Second, the majority of included studies were cross-sectional and relied on self- or parent-report measures, with limited use of objective mobility tools, which constrains causal inference and may miss multimodal and partial-active trips. Third, the coverage of social determinants (e.g., parental work schedules, independent mobility norms) was uneven. Fourth, national analyses that classify active school travel as a single predominant mode may undercount partial-active commutes via public transport, suggesting that surveillance should capture multimodal trips common in Chinese contexts. Finally, screening and data extraction were conducted by a single reviewer, with verification of a 20% random subsample to mitigate potential selection and extraction bias.

## 5 Conclusion

This scoping review maps the Chinese evidence on AT in children and adolescents and finds a small but growing, fragmented literature dominated by cross-sectional, self-reported studies. AT appears to contribute meaningfully to daily physical activity, with after-school trips providing notable increments in urban settings. Individual, parental, and built-environment features consistently shape mode choice, including distance, safety perceptions, and street connectivity. Although intervention evidence within China remains sparse, broader systematic reviews indicate that multicomponent programs and infrastructure changes can shift mode share and deliver co-benefits beyond physical activity. Future studies should prioritize longitudinal and quasi-experimental designs, standardized and device-based measures, and attention to equity and mental health outcomes to produce decision-ready evidence for policy and practice.
