# Supplementary material for: A scoping review of children’s and adolescents’ active travel in China
Source: Front Public Health. 2026 Aug 5;14:1771411. doi: 10.3389/fpubh.2026.1771411 (PMC13488422; doi:10.3389/fpubh.2026.1771411)
Supplement: Supplementary file 2 [file Table_2.docx]

**Keywords:**

*China*

*Chinese*

*walk*

*walking*

*run**

*running*

*bike*

*bicycle*

*bicycling*

*cycle*

*cycling*

*stroll*

*strolling*

*ride*

*riding*

*active transport*

*active transportation*

*active transit*

*active transporting*

*active commut**

*travel mode*

*mode of travel**

*active travel*

*active travelling*

*travel mode*

*trip*

*school travel*

*non-motori*ed*

Timespan: From inclusion to 2025-12-01

**Web of science**

("China" OR "Chinese") (Title) and ("walk" OR "walking" OR "run*" OR "running" OR "bike" OR "biking" OR "bicycle" OR "bicycling" OR "cycle" OR "cycling" OR "stroll" OR "strolling" OR "ride" OR "riding" OR "active transport" OR "active transportation" OR "active transit" OR "active transporting" OR "active commut*" OR "travel mode" OR "mode of travel*" OR "active travel" OR "active travelling" OR "travel mode" OR "trip" OR "school travel" OR "non-motori*ed") (Title)

**PubMed**

(("China"[Title] OR "Chinese"[Title])) AND (("walk"[Title] OR "walking"[Title] OR "run*"[Title] OR "running"[Title] OR "bike"[Title] OR "biking"[Title] OR "bicycle"[Title] OR "bicycling"[Title] OR "cycle"[Title] OR "cycling"[Title] OR "stroll"[Title] OR "strolling"[Title] OR "ride"[Title] OR "riding"[Title] OR "active transport"[Title] OR "active transportation"[Title] OR "active transit"[Title] OR "active transporting"[Title] OR "active commut*"[Title] OR "travel mode"[Title] OR "mode of travel*"[Title] OR "active travel"[Title] OR "active travelling"[Title] OR "travel mode"[Title] OR "trip"[Title] OR "school travel"[Title] OR "non-motori*ed"[Title]))

**SportDiscus**

TI (("China" OR "Chinese")) AND TI (("walk" OR "walking" OR "run*" OR "running" OR "bike" OR "biking" OR "bicycle" OR "bicycling" OR "cycle" OR "cycling" OR "stroll" OR "strolling" OR "ride" OR "riding" OR "active transport" OR "active transportation" OR "active transit" OR "active transporting" OR "active commut*" OR "travel mode" OR "mode of travel*" OR "active travel" OR "active travelling" OR "travel mode" OR "trip" OR "school travel" OR "non-motori*ed"))

**MEDLINE**

TI (("China" OR "Chinese")) AND TI (("walk" OR "walking" OR "run*" OR "running" OR "bike" OR "biking" OR "bicycle" OR "bicycling" OR "cycle" OR "cycling" OR "stroll" OR "strolling" OR "ride" OR "riding" OR "active transport" OR "active transportation" OR "active transit" OR "active transporting" OR "active commut*" OR "travel mode" OR "mode of travel*" OR "active travel" OR "active travelling" OR "travel mode" OR "trip" OR "school travel" OR "non-motori*ed"))

**ERIC**

TI (("China" OR "Chinese")) AND TI (("walk" OR "walking" OR "run*" OR "running" OR "bike" OR "biking" OR "bicycle" OR "bicycling" OR "cycle" OR "cycling" OR "stroll" OR "strolling" OR "ride" OR "riding" OR "active transport" OR "active transportation" OR "active transit" OR "active transporting" OR "active commut*" OR "travel mode" OR "mode of travel*" OR "active travel" OR "active travelling" OR "travel mode" OR "trip" OR "school travel" OR "non-motori*ed"))
